# Characteristics of severely malnourished under-five children immunized with Bacillus Calmette-Guérin following Expanded Programme on Immunization schedule and their outcomes during hospitalization at an urban diarrheal treatment centre, Bangladesh

**DOI:** 10.1371/journal.pone.0262391

**Published:** 2022-01-07

**Authors:** Mst. Mahmuda Ackhter, Abu Sadat Mohammad Sayeem Bin Shahid, Tahmeed Ahmed, Parag Palit, Irin Parvin, Md. Zahidul Islam, Tahmina Alam, Shamsun Nahar Shaima, Lubaba Shahrin, Farzana Afroze, Monira Sarmin, Shoeb Bin Islam, Zubair Akhtar, Mohammod Jobayer Chisti, Fahmida Chowdhury

**Affiliations:** 1 Nutrition and Clinical Services Division, International Centre for Diarrhoeal Disease Research, Bangladesh (icddr,b), Dhaka, Bangladesh; 2 Infectious Diseases Division, International Centre for Diarrhoeal Disease Research, Bangladesh (icddr,b), Dhaka, Bangladesh; The University of Georgia, UNITED STATES

## Abstract

**Background:**

Bacillus Calmette-Guérin (BCG) vaccination has recently been found to have beneficial effects among children infected other than *Mycobacterium tuberculosis*. Due to the paucity of data on the outcomes of children who had successful BCG vaccination following Expanded Programme on Immunization (EPI) schedule, we aimed to investigate the characteristics of such children and their outcomes who were hospitalized for severe malnutrition.

**Methods:**

A prospective observational study was conducted to determine the viral etiology of pneumonia in severely malnourished children those were admitted to the Dhaka Hospital of International Centre for Diarrhoeal Disease Research, Bangladesh (icddr,b) between April 2015 and December 2017, constituted the study population. Using a case-control design for the analysis, children having BCG vaccination prior hospital admission were treated as cases (n = 611) and those without vaccination, constituted as controls (n = 83). Bi-variate analysis was conducted using socio-demographic, clinical, laboratory, and treatment characteristics on admission and outcomes during hospitalization. Finally, log-linear binomial regression analysis was done to identify independent impact of BCG vaccination.

**Results:**

The cases more often presented with older age, have had lower proportion of maternal illiteracy, higher rate of breastfeeding, severe wasting and lower rate of hypoglycemia, compared to the controls. The cases were also found to have lower risk of severe sepsis and deaths, compared to the controls (for all, p<0.05). However, in log-linear binomial regression analysis, after adjusting for potential confounders, BCG vaccination following EPI schedule (RR:0.54; 95%CI = 0.33–0.89; p = 0.015) and breastfeeding (RR:0.53; 95%CI = 0.35–0.81; p = 0.003) were found to be protective for the development of severe sepsis.

**Conclusion:**

BCG vaccination and breastfeeding were found to be protective for the development of severe sepsis in hospitalized severely malnourished under-five children which underscores the importance of continuation of BCG vaccination at birth and breastfeeding up to two years of age.

## Introduction

The Bacillus Calmette-Guérin (BCG) vaccine, initially developed attenuating a strain of *Mycobacterium bovis* [1] is one of the oldest and most commonly administered vaccines worldwide. Albert Calmette and Camille Guérin, two pioneer scientists invented the vaccine in early 1900s by attenuating a strain of *Mycobacterium bovis that was* closely related to *Mycobacterium tuberculosis*, causing bovine tuberculosis (TB) [1]. The World Health Organization (WHO) implemented Expanded Programme on Immunization (EPI) in Bangladesh on 1979 having its lower impact until 1985. Later on, government took an initiative to improve childhood vaccination coverage from 1985 onwards. The United Nations Children’s Fund and the WHO estimated that individual vaccine coverage in Bangladesh in 2015 for BCG was 98% [2]. Although BCG vaccine is intended to prevent tuberculosis, it may also have some non-specific benefits, potentially in reducing morbidity and mortality [3]. Historical data as well as data from an observational study conducted in low and middle income countries by Roth et al, [4–8] have suggested that BCG vaccine may have some nonspecific beneficial effects on child survival [9].

BCG induces functional changes in the innate and adaptive immune compartments in the first year of life. Understanding the biological mechanisms beyond its heterogeneous effects is crucial to improve the protection that the vaccine confers to the infants from infectious diseases [10]. Some human and animal studies suggested that priming with one pathogen may trigger innate immune responses or induce heterologous T-cell mediated immunity, thus reducing the susceptibility to subsequent infections with *Mycobacterium tuberculosis* and thus also proving that BCG vaccine has its non-specific effects [11–14]. In the neonatal period, BCG vaccine has been reported to induce strong Th1 responses [15] and also stimulate Th1 and Th17 responses to non-mycobacterial pathogens [16–18]. Findings from two separate studies also concluded that BCG vaccination protects against non-mycobacterial infections, especially against sepsis and other respiratory infections [19,20]. WHO’s Strategic Advisory Group of Experts on Immunization also concluded that BCG and measles vaccine may have beneficial effects and advocated for further research to address this specific issue [21].

Malnutrition accounts for 35% of all cases of morbidities among children less than five years of age and for around 3.5 million cases of annual global mortality, thereby distinctly indicating that malnutrition, a salient cause of life threatening conditions among such children in developing countries [22]. In Bangladesh, prevalence of under-five stunting, wasting and underweight are 36%, 14% and 33%, respectively [23]. A prospective study conducted by Chisti et al, from April 2011 to June 2012 concluded that severely malnourished children are also commonly affected with TB, especially in TB endemic areas, like Bangladesh [24].

Following introduction of BCG vaccine in the 1920’s, experts in the field of infectious diseases had been in concurrence that BCG vaccine had intermittent heterogeneous protective effects on childhood morbidity and mortality beyond the spectrum of the particular protection against tuberculosis [25]. Considering the non-specific effects of BCG vaccination and the burden of TB among severely malnourished under-five children in Bangladesh, this study aimed to evaluate the characteristics and outcomes of BCG vaccinated children.

## Materials and methods

### Ethical consideration

This study (PR-15011) was approved by the institutional review board (comprised of research review committee and ethical review committee) of International Centre for Diarrhoeal Disease Research, Bangladesh (icddr,b). Written, informed consent was obtained from the caregivers before enrolling the children in this study.

### Study setting and design

The study was carried out in the Dhaka Hospital of icddr,b. Each year, the hospital offers free of cost treatment to around 150,000 patients, among whom 60% are children under the age of five years admitted with history of diarrhea and or acute respiratory infections and other associated complications. The vast majority of the patients live in urban and peri-urban Dhaka city and belong to poor socio-economic background.

**Fig 1:**
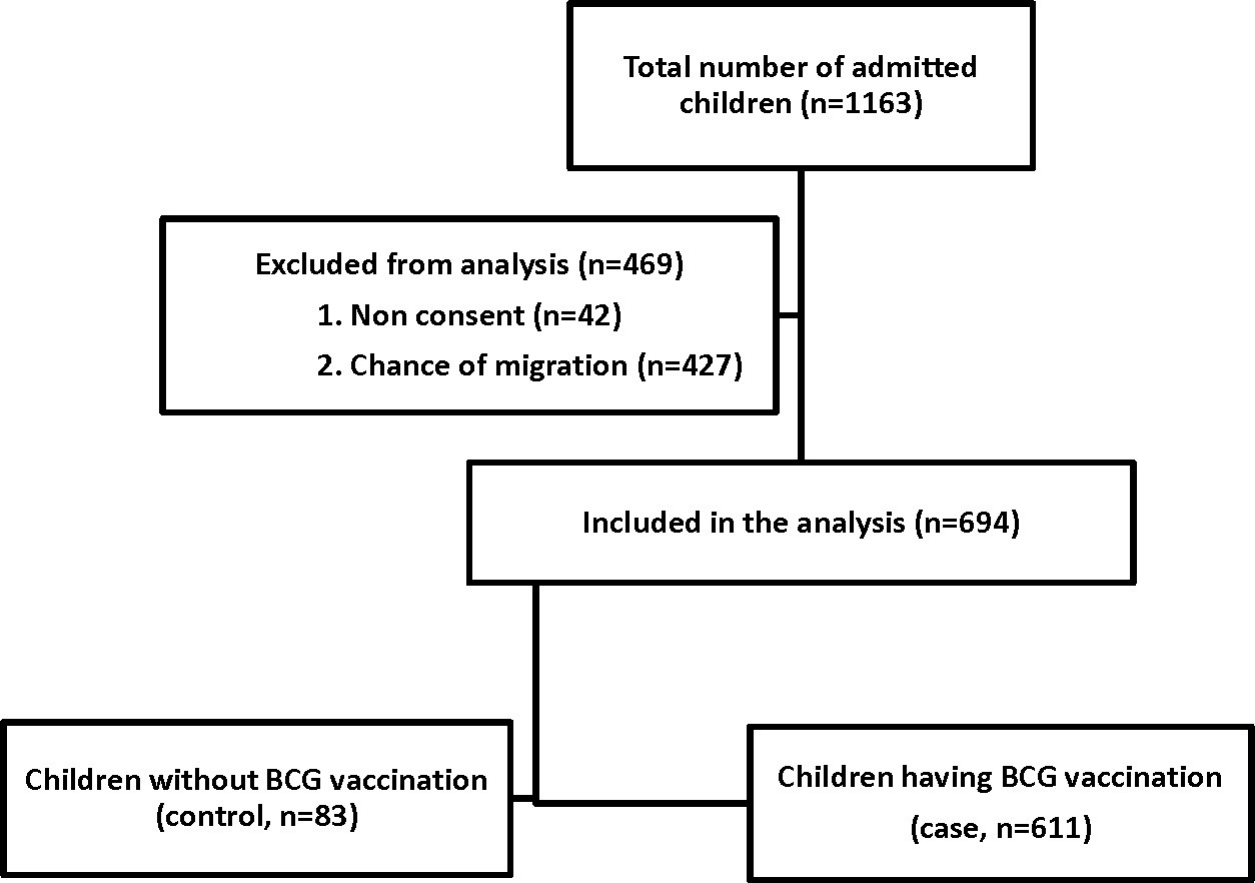
Study profile.

A total of 1163 under-five children suffering from severe malnutrition was screened under a prospective observational study conducted to evaluate the viral etiology of pneumonia. Among them, 694 children met the inclusion criteria (severely malnourished under-five children of either sex admitted with acute illnesses, like diarrhea, pneumonia). We excluded 469 children due to non-consent and chance of migration leading to lost to follow up in that prospective study (Fig 1). By analyzing the data using a case control design, where children having BCG vaccination prior hospital admission were considered as cases (n = 611) and those without BCG vaccination were considered as controls (n = 83). Comparison of socio-demographic, clinical, laboratory, and treatment characteristics on admission and their outcomes during hospitalization was carried out between the groups.

### Case definition

Severe malnutrition: WHO criteria <-3 z score from the median of weight for height/length, weight for age, or nutritional edema [26].

Paternal and maternal illiteracy: Parents who did not attend formal schooling [26].

Dehydration (some/severe): Defined by the Dhaka method, which is almost similar to the WHO method and approved by that organization [27].

Pneumonia: If a child presents with severe malnutrition with any sign of pneumonia (any of the WHO defined signs of pneumonia or severe pneumonia or radiological pneumonia) would be considered as pneumonia [28].

Severe wasting: z-score for weight for length/height <-3 of the WHO growth standard [29].

Severe underweight: z-score for weight for age <-3 of the WHO growth standard [29].

Severe sepsis: Defined as sepsis with sepsis-induced organ dysfunction or tissue hypo-perfusion [30].

Heart failure: Defined as a clinical and pathophysiologic syndrome that results from ventricular dysfunction, volume, or pressure overload, alone or in combination [31].

Respiratory failure: Defined as the presence of any one of the following phrases: mechanical ventilation, intubation, oxygen, continuous positive airway pressure (CPAP), bubble CPAP, nasal cannula, or ventilator [32].

Hypoglycemia: random blood sugar <3.0 mmol/L [33].

Abnormal C-reactive protein: ≥ 1 mg/dl [33].

Hyponatremia: serum sodium <130.0 mmol/L [33].

Hypernatremia: serum sodium >150.0 mmol/L [34].

Hypokalemia: serum potassium <3.5 mmol/L [33].

### Patient management

Antibiotics and other supportive care were provided in line with the hospital’s treatment protocol for severe malnutrition (S1 Appendix). Trained study physicians followed up the patients and recorded their clinical parameters daily in the electronic database. Treatment changes were performed based upon either clinical deterioration or non-improvement of clinical findings after 48 hours following hospitalization. If patients required treatment for health-related complications that was not available at the hospital, like renal dialysis or any kind of surgical interventions, they were immediately referred to other specialized hospitals designated for such treatments.

### Measurements

Trained study physicians and research assistants collected data from caregivers during admission and filled in specially designed and well-structured case report forms.

Data on socio-demographic and vaccination status was collected. Clinical characteristics included the presence of fever, diarrhea, dehydration (some/severe), pedal edema, pneumonia, severe wasting, and severe underweight. The laboratory tests variables assessment for hypoglycemia, abnormal C-reactive protein levels, hypokalemia, hyponatremia, hypernatremia, positive bacterial culture and viral polymerase chain reaction (PCR) during hospitalization was performed (Table 1). Treatment characteristics of both the groups were also collected and recorded during hospitalization (Table 2). Outcome characteristics were severe sepsis, heart failure, respiratory failure, and death during hospitalization and also duration of hospital stay (Table 3). Finally, log-linear binomial regression analysis was done to see the independent impact of BCG vaccination.

**Table 1:** Comparison of socio-demographic, clinical and laboratory characteristics of severely malnourished under-five children with (cases) and without (control) BCG vaccination on admission.

Characteristics	Children immunized with BCG vaccine (Case, n = 611)	Children not immunized with BCG vaccine (Control, n = 83)	OR	95% CI	p-value
Age in months (median, IQR)	9.8 (5.6, 16.0)	3.5 (2.6, 7.7)	-	-	<0.001
Male sex	377 (62)	50 (60)	1.06	0.66–1.70	0.797
Illiterate father	153 (25)	19 (23)	1.12	0.65–1.94	0.670
Illiterate mother	129 (21)	31 (37)	0.45	0.28–0.73	0.001
Caregivers monthly income in BDT (median, IQR)	10000 (7500, 15000)	9000 (7000, 15000)	-	-	0.268
Residing in slum	65 (11)	11 (13)	0.78	0.39–1.54	0.474
Breast feeding (exclusive/partial)	457 (75)	39 (47)	3.35	2.09–5.34	<0.001
Presence of diarrhea on admission	535 (88)	73 (88)	0.96	0.48–1.95	0.919
Presence of fever on admission	213 (35)	31 (37)	0.90	0.56–1.44	0.656
Presence of pedal edema on admission	43 (7)	10 (12)	0.55	0.27–1.15	0.107
Presence of dehydration (some/severe) on admission	94 (18)	17 (23)	0.70	0.39–1.26	0.236
Presence of both clinical and radiological pneumonia	214 (35)	34 (41)	0.83	0.42–1.63	0.555
Severe wasting (WLZ<-3 SD)	357 (58)	33 (40)	2.13	1.33–3.40	0.001
Severe underweight (WAZ<-3 SD)	579 (95)	77 (93)	1.41	0.57–3.48	0.454
Presence of hypoglycemia (random blood glucose<3 mmol/L) on admission	9/597 (2)	5/79 (6)	0.23	0.07–0.69	0.016
Abnormal C-reactive protein (≥1.0 mg/L) on admission	168/313 (54)	29/47 (62)	0.72	0.38–1.35	0.303
Presence of hypokalemia (K<3.5 mmol/L) on admission	82/343 (24)	14/54 (26)	0.90	0.46–1.73	0.747
Presence of hyponatremia (Na<130 mmol/L) on admission	35/343 (10)	6/54 (11)	0.91	0.36–2.28	0.839
Presence of hypernatremia (Na>150 mmol/L) on admission	43/343 (13)	10/54 (19)	0.63	0.29–1.34	0.230
Positive bacterial culture	13/318 (4)	3/48 (6)	0.64	0.17–2.33	0.351
Positive viral PCR	330/608 (54)	50/81 (62)	0.74	0.46–1.18	0.205

n = number; parenthesis at the right side of the dichotomous variable denotes ‘%’ unless specified otherwise; IQR, inter-quartile range; BDT, Bangladeshi taka; SD, standard deviation; PCR, polymerase chain reaction; OR, odds ratio; CI, confidence interval.

**Table 2:** Comparison of treatment characteristics of severely malnourished under-five children with (cases) and without (control) BCG vaccination during hospitalization.

Characteristics	Children immunized with BCG vaccine (Case, n = 611)	Children not immunized with BCG vaccine (Control, n = 83)	OR	95% CI	p-value
Received Ampicillin during hospitalization	543 (89)	70 (84)	1.48	0.78–2.82	0.227
Duration of Ampicillin in days (median, IQR)	3.0 (3.0, 4.0)	3.0 (2.0, 5.0)	-	-	0.693
Received Gentamicin during hospitalization	546 (89)	70 (84)	1.56	0.82–2.97	0.174
Duration of Gentamicin in days (median, IQR)	5.0 (3.0, 7.0)	4.0 (2.0, 7.0)	-	-	0.087
Received Ceftriaxone during hospitalization	164 (27)	25 (30)	0.85	0.52–1.41	0.529
Duration of Ceftriaxone in days (median, IQR)	6.0 (4.0, 7.0)	7.0 (3.0, 10.0)	-	-	0.500
Received Levofloxacin during hospitalization	157 (26)	24 (29)	0.85	0.51–1.41	0.531
Duration of Levofloxacin in days (median, IQR)	6.0 (4.0, 7.0)	7.0 (3.0, 8.5)	-	-	0.852
Received Ceftazidime during hospitalization	60 (10)	9 (11)	0.89	0.43–1.88	0.770
Duration of Ceftazidime in days (median, IQR)	7.0 (6.0, 9.5)	7.0 (2.0, 8.0)	-	-	0.590
Received Amikacin during hospitalization	56 (9)	6 (7)	1.29	0.54–3.11	0.886
Duration of Amikacin in days (mean, SD)	7.5±3.4	7.3±4.4	-	-	0.829

n = number; parenthesis at the right side of the dichotomous variable denotes ‘%’ unless specified otherwise; IQR, inter-quartile range; SD, standard deviation; OR, odds ratio; CI, confidence interval.

**Table 3:** Outcomes of severely malnourished under-five children with (cases) and without (control) BCG vaccination during hospitalization.

Characteristics	Children immunized with BCG vaccine (Case, n = 611)	Children not immunized with BCG vaccine (Control, n = 83)	RR	95% CI	p-value
Severe sepsis during hospitalization	60 (10)	20 (24)	0.41	0.26–0.64	<0.001
Heart failure during hospitalization	30 (5)	5 (6)	0.82	0.32–2.04	0.665
Respiratory failure during hospitalization	18 (3)	4 (5)	0.61	0.21–1.76	0.362
Mechanical ventilation during hospitalization	5 (1)	1 (1)	0.68	0.08–5.75	0.722
Duration of hospitalization in days (median, IQR)	6.0 (4.0, 10.0)	5.0 (3.0, 8.0)	-	-	0.210
Death	29 (5)	10 (12)	0.39	0.20–0.78	0.006

n = number; parenthesis at the right side of the dichotomous variable denotes ‘%’ unless specified otherwise; RR, relative risk; CI, confidence interval; IQR, inter-quartile range.

### Statistical analysis

Data was entered using SPSS for Windows version 20.0 (SPSS Inc, Chicago, IL), and analyzed using STATA version 13 (College Station, Texas). For qualitative variables, differences in proportions were compared by the Chi-square or Fisher’s exact test, where appropriate. Differences in means for normally distributed quantitative data were compared by the Student’s *t*-test and the Mann-Whitney U test was used for comparison of non-parametric data. A probability of less than 0.05 was considered statistically significant. The strength of association was determined by calculating the odds ratio (OR) or relative risk (RR), as appropriate and their 95% confidence intervals (CIs). In the bi-variate model, socio-demographic, clinical and laboratory characteristics that were analyzed during admission included age, male sex, paternal and maternal illiteracy, caregiver’s monthly income, residence, breastfeeding history, fever, diarrhea, dehydration status (some/severe), pedal edema, pneumonia, severe wasting, severe underweight, hypoglycemia, abnormal C-reactive protein levels, hypokalemia, hyponatremia, hypernatremia, positive bacterial culture and viral PCR. In another bi-variate analysis, outcome characteristics analyzed included severe sepsis, heart failure, respiratory failure, and death as well as duration of hospital stay. Finally, log-linear binomial regression analysis was performed to identify whether BCG vaccination had independent association with worse outcomes, such as severe sepsis, after adjusting with potential confounders.

## Results

1163 children, aged 0–59 months admitted to the Dhaka Hospital’s intensive care unit or longer stay unit with severe malnutrition during the study period. We analysed data of 694 children out of 1163, after obtaining informed written consent from parents/caregivers (Fig 1). Among them, 611 were cases and 83 were controls. During admission, the cases presented with older age, have had lower proportion of maternal illiteracy, higher rate of breastfeeding, severe wasting and lower rate of hypoglycemia, compared to the controls (Table 1). Other characteristics, like positive bacterial culture and viral PCR were comparable between the groups (Table 1). Treatment characteristics among the groups were also comparable (Table 2). In another bi-variate analysis, the cases were found to be significantly associated with lower risk of severe sepsis and deaths, compared to the controls (Table 3). In log-linear binomial regression analysis, after adjusting for potential confounders, age, BCG vaccination following EPI schedule and breastfeeding remained to be protective for the development of severe sepsis (Table 4). When we kept death as dependent variable in another regression model, we didn’t find any significant association of BCG vaccination with death (RR: 0.86; 95%CI = 0.43–1.72; p = 0.679).

**Table 4:** Results of log-linear binomial regression analysis to explore the independently associated factors with severe sepsis in severely malnourished under-five children.

	Un-adjusted			Adjusted		
Characteristics	RR	95% CI	p-value	RR	95% CI	p-value
BCG vaccination	0.83	0.73–0.95	0.006	0.54	0.33–0.89	0.015
Age	0.96	0.93–0.98	0.002	0.97	0.95–0.99	0.046
Illiterate mother	1.03	0.68–1.57	0.874	0.97	0.59–1.57	0.891
Breastfeeding	0.71	0.57–0.88	0.001	0.53	0.35–0.81	0.003
Severe wasting	0.95	0.77–1.18	0.647	1.11	0.73–1.69	0.634

RR, relative risk; CI, confidence interval.

## Discussion

The main observation of this study was the protective effect of BCG vaccination following EPI schedule and breastfeeding for the development of severe sepsis during hospitalization of severely malnourished under-five children.

The observation of heterogenous protective effect of BCG vaccination for development of severe sepsis is explicable. Cell mediated and humoral immunity is usually depressed in severely malnourished children making them highly vulnerable to infectious diseases. In a recent study, lack of BCG vaccination was found to be one of the predicting factors for severe sepsis among severely malnourished under-five children hospitalized with pneumonia [35], which corroborates with the findings of this study.

The beneficial effects of BCG vaccination on non-tubercular illness have been well documented. BCG vaccination has been reported to reduce around 50% of deaths from non-tubercular infections in developing countries with high childhood mortality. This may reflect growing evidence on substantial heterogeneous effects of BCG vaccination in children, including its potentials for reducing the incidence of severe sepsis in TB endemic developing countries, like Bangladesh. This carries an important message for the clinicians as well as policymakers, especially in developing countries, where severe sepsis related deaths are high, for promoting BCG vaccination [36,37].

We also observed the protective effect of breastfeeding to halt severe sepsis in our study children. Severely malnourished children who were non-breastfed at their neonatal period, were more prone to develop severe sepsis compared to breastfed children which signifies the importance of continuation of breastfeeding in infancy and supports our study findings [35]. In addition to breast milk’s nutritional advantages, it reduces frequency of infections, particularly gastrointestinal [38,39] and respiratory tract [39–42] through specific and non-specific immune factors [43]. A study conducted in Ethiopia showed breastfeeding might reduce mortality by 59% among breastfed under-five SAM children in comparison to those who were non-breastfed [44].

We also observed the protective effect of older age for the development of severe sepsis in our study that might be due to potentially having better immunity of older children than their younger counterpart [45].

In the bi-variate analysis, we also observed lower risk of death in our study children immunized with BCG, although the association became insignificant after adjusting for potential confounders. However, a number of previous studies revealed the protective effect of BCG vaccination in reducing deaths. Findings from a study conducted by Chisti et al, showed lack of BCG vaccination to be an independent predictor of bacteremia leading to mortality among severely malnourished children aged less than 5 years of age [46]. Study conducted by Roy P et al, showed administration of BCG vaccine at birth reduces all-cause mortality for its non-specific beneficial effects, in addition to its beneficial effect on reduction of mortality from tuberculosis [47]. Another study conducted by Hervie S et al, also came up with the same result [48]. Ritz et al, concluded that BCG vaccine had heterogeneous beneficial impact and influence the antibody response when administered at birth which may lead to reduce mortality in late infancy [49]. Study conducted by Nankabirwa V et al, showed children vaccinated with BCG had lower mortality, compared to non-vaccinated children [50].

The major strength of our study was that it aimed to determine the heterogeneous impact of BCG vaccination prior hospital admission in severely malnourished under-five children that was unique in the context of clinical research in this particular field. Another strength of the study was its design, which helped to ensure minimum statistical errors during the analysis. However, a major limitation of this study was involved with an overall comparatively low and mismatched sample size between the cases and the controls, thus providing a possible confounding effect in drawing pertinent statistical inference.

## Conclusion

The results of our study helped us to draw an inference that BCG vaccination following EPI schedule and breastfeeding were found to be protective for the development of severe sepsis among children hospitalized for severe malnutrition. The observation underscores the importance of scrupulously adhering to EPI guidelines for continuation of BCG vaccination in order to reduce the risk of non-tubercular illness, such as severe sepsis that may lead to reduced mortality during hospitalization among severely malnourished children, especially in TB endemic countries. It is more likely that timely BCG vaccination is a marker of better clinical care early in life and possibly more of a marker of social determinants of health. Further research with a larger sample is imperative to define the path towards obtaining unequivocal evidence on these issues that would support future robust, evidence-based adjustments in immunization policies, especially in resource constrained settings.

## Supporting information

S1 Appendix (DOCX)

## Abbreviations

BCG: Bacillus Calmette-Guerin
WHO: World Health Organization
EPI: Expanded Programme on Immunization
icddr,b: International Centre for Diarrhoeal Disease Research, Bangladesh
