# Supplementary material for: Characteristics of severely malnourished under-five children immunized with Bacillus Calmette-Guérin following Expanded Programme on Immunization schedule and their outcomes during hospitalization at an urban diarrheal treatment centre, Bangladesh
Source: PLoS One. 2022 Jan 7;17(1):e0262391. doi: 10.1371/journal.pone.0262391 (PMC8741016; doi:10.1371/journal.pone.0262391)
Supplement: S1 Appendix — (DOCX) [file pone.0262391.s001.docx]

**S1 Appendix**

**Management of severe acute malnutrition (SAM)**

**1. Dietary therapy**

Feeding needed to be started on admission or within two hours of starting rehydration for children with dehydration. Mothers were advised to continue breastfeeding, if applicable. Feeding was started with a liquid diet, such as milk suji/F75 (10 ml/kg per feed), given every two hours (12 feeds per day), providing about 80 kcal/kg per day. As the child's appetite improved, the amount of feed were increased so that a calorie intake of 100 kcal/kg per day and protein intake of 1-1.5 g/kg per day was achieved. Energy intake was only increased after the child's general condition had improved and their appetite had returned. If the child had a poor appetite, could not take food orally due to weakness or painful mouth/tongue sores, or had a rapid respiratory rate, a nasogastric (NG) tube was introduced. Feeds were offered orally, and the amount not taken orally will be given through NG tube. The NG tube will be withdrawn If the child can take at least three-fourths of the diet orally or take two consecutive feeds. It may reintroduced, if intake fell below 80 kcal/kg per day over the next few days.

**2. Antibiotic treatment**

Ampicillin (100 mg/kg per day in six-hourly doses) and Gentamicin (5 mg/kg per day in 12-hourly doses) were administered intravenously for five days for children without pneumonia. The dose of ampicillin was increased to 200 mg/kg per dayand antibiotic therapy was continued for 7-10 days in the following cases: if there was no clinical improvement within 48 hours (in terms of fever response, respiratory rate, or if the pulse becomes weak), if the clinical condition deteriorated after 24 hours of treatment or if septicemia was suspected (presence of a weak pulse, hypothermia, hypoglycemia, or shock in the absence of dehydration). Ceftriaxone, if available, was given 100mg/kg once daily instead of ampicillin, along with gentamicin, in cases of septic shock. As per the World Health Organization (WHO) and Dhaka Hospitalof International Centre for Diarrhoeal Disease Research, Bangladesh (icddr,b) guidelines, children with severe pneumonia were treated with ceftriaxone and levofloxacin as second-line, if the first-line treatment of ampicillin and gentamicin failed after 48-72 hours of therapy. Ceftazidime and amikacin was the third-line choice for therapy for severe pneumonia, based on the etiology, in severely malnourished children. Children, who did not have any apparent complications and were managed on a daycare basis, were treated with syrup. amoxycillin (50 mg/kg per day in eight-hourly doses) for five days.

**3. Vitamins and mineral supplements**

a. Vitamin A: If there was no xerophthalmia (eye diasese due to vitamin A deficiency), vitamin A was given at 200,000 units above one year of age, 100,000 units for infants aged 6-12 months, and 50,000 units for those below six months of age.

b. Zinc: Elemental zinc, at 2 mg/kg per day, was given for at least one month.

c. Multivitamins: Multivitamin drops been provided for at least one month at a dose of 1 ml twice daily for children aged one year plus and 0.5 ml twice daily for infants under one year of age.

d. Folic acid: A quarter of a 5 mg/tablet was given once daily for at least 15 days.

e. Potassium: Potassium solution was given by mouth at a dose of 4 mmol/kg per day, three times daily, for five days (5 ml of commercially available potassium chloride syrup contains seven mmol of elemental potassium).

f. Magnesium: An intramuscular injection of magnesium sulfate (50%) 0.1 ml/kg (0.4 mmol/kg) was given once daily for seven days.

**4. Correcting dehydration**

If any child had some dehydration, oral rehydration solution (ORS) was given at 10 ml/kg per hour for the first two hours and then 5 ml/kg per hour for the next 10 hours or until the fluid deficit was corrected. Besides, ongoing stool losses were replaced with 5-10 ml/kg of ORS after each watery or loose stool. The mother was always be instructed about the quantity of ORS to be given in terms of teaspoons per hour (e.g., 40 ml/hour = eight teaspoons/hour). ORS was administered through naso-gastric (NG) tube, if a child was unable to drink due to weakness or vomiting. If the child had some dehydration but required intravenous (IV) fluids because of vomiting that had continued even after introducing a NG tube or because of severe abdominal distension, IV 1/2 strength cholera saline with 5% dextrose was given @10 ml/kg per hour for the first two hours, then 5 ml/kg per hour till the fluid deficit was corrected, or when the child became able to take ORS. If possible, extra potassium was added to the infusion (injection potassium chloride,13 mmol/L of infusion). For children with severe dehydration (fluid deficits of 10% or more of body weight), initial hydration was done with IV cholera saline with 5% dextrose. If possible, 7 mmol of injection potassium chloride solution was added to each liter of the infusion. The IV fluid was given at the following rates: first hour: 20 ml/kg, second hour: 10 ml/kg. The total fluid required was calculated, and the prescription was given in terms of drops per minute (1 ml = 60 drops, by a micro drip or soluset). For infants under two months, 1/2 strength cholera saline with 5% dextrose was used in place of full-strength cholera saline. If possible, 13 mmol of injection potassium chloride was added to one liter of the infusion. At the end of the first hour, ORS was provided @ 10 ml/kg per hour. At the end of second hour, the IV needed to be discontinued. ORS was continued @ 10 ml/kg per hour for two hours, then slowed down to 5 ml/kg per hour for the next 10 hours or until the fluid deficit was corrected. If the child was unable to drink due to weakness or vomiting, ORS was administered through NG tube .
